# Size-fractioned ultrafine particles and black carbon associated with autonomic dysfunction in subjects with diabetes or impaired glucose tolerance in Shanghai, China

**DOI:** 10.1186/s12989-015-0084-6

**Published:** 2015-03-25

**Authors:** Yitong Sun, Xiaoming Song, Yiqun Han, Yunfang Ji, Shuna Gao, Yu Shang, Shou-en Lu, Tong Zhu, Wei Huang

**Affiliations:** Department of Occupational & Environmental Health Sciences, and Institute of Environmental Medicine, Peking University School of Public Health, and Key Laboratory of Molecular Cardiovascular Sciences, Ministry of Education, Beijing, 100191 China; College of Environmental Sciences and Engineering, Peking University, Beijing, China; Center for Diseases Control and Prevention of Luwan District, Shanghai, China; Institute of Environmental Pollution and Health, Shanghai University, Shanghai, China; Department of Biostatistics, UMDNJ-School of Public Health, Piscataway, New Jersey US

**Keywords:** Ultrafine particulates, Black carbon, Traffic pollution, Heart rate variability, Diabetes, Impaired glucose tolerance

## Abstract

**Background:**

Particles in smaller size fractions, such as ultrafine particles (UFPs) (with diameter less than 100 nm), has become of significant cardiovascular health concerns. However, the biological plausibility underlying potential relationship between UFPs and cardiovascular outcomes is less studied.

**Methods:**

Fifty-three subjects living in Shanghai with type-2 diabetes (T2D) or impaired glucose tolerance (IGT) were followed for autonomic dysfunctions with three repeated measurements in 2010. Minute-to-minute concentrations of ambient particles in small size-fractions (5-560 nm), black carbon (BC), sulfur dioxide (SO _2_), nitrogen dioxide (NO_2_), carbon monoxide (CO), and ozone (O_3_) were monitored using a central monitoring laboratory equipped with real-time air monitors close to residential area of the subjects. Generalized linear mixed models, with adjustment for individual risk factors, were applied to assess the effects of air pollution on autonomic dysfunctions in subjects.

**Results:**

Our study showed that significant reduction in the standard deviation of all NN intervals (SDNN) ranging from 3.4% to 8.1% were associated with interquartile range (IQR) increase of number concentration of particles (PNC) in size fractions <100 nm, and reduction from 1.3% to 4.6% with particles of diameter 100-200 nm, in subjects with diabetes or glucose tolerance. Increased exposure to traffic-related pollutants BC, NO_2_ and CO, and combustion pollutant SO_2_, were also significantly associated with HRV reductions. However, no effect was observed for particles in size fraction of 200-560 nm and O_3_. Diabetic risk factor and gender appeared to have significant interactions on autonomic dysfunction associated with UFPs and traffic pollution exposures in certain time-window.

**Conclusions:**

Our results suggest that underlying diabetes or impaired glucose tolerance may confer reduced autonomic function of heart due to traffic pollution exposure.

**Electronic supplementary material:**

The online version of this article (doi:10.1186/s12989-015-0084-6) contains supplementary material, which is available to authorized users.

## Background

Association between particulate matter, with diameter less than 10 and 2.5 μm (PM_10_ and PM_2.5_), and cardiovascular diseases (CVD) has been supported by numerous epidemiologic studies [1]. Particles in smaller size fractions, such as ultrafine particles (UFPs) (with diameter less than 100 nm), have have been reported that may contribute to increasing cardiovascular morbidity [2-4]. Particles emitted from transportation and industry combustion have been found dominating the number and surface concentrations of ambient particulate matters in small size fractions [4-6], and carrying large amount of adsorbed or condensed toxic air pollutants (oxidant gases, organic compounds, and transition metals) [7]. Studies reported that UFPs can potentially lead to acute cardiovascular responses [8-11]. However, compared to ambient PM_2.5_ and PM_10_, underlying biological plausibility of relationship between UFPs and cardiovascular morbidity has been far less studied.

Epidemiologic study findings suggested altered heart rate variability (HRV) as a possible mechanism linking ambient PM exposure to coronary heart disease events in subjects with preexisting cardiometabolic diseases [12-16]. Reduced HRV has been found associated with increasing risk of first cardiovascular event in population without known cardiovascular diseases [17], and associated with the development of coronary heart disease in individuals with diabetes [18]. The reduction of HRV derived from electrocardiographic (ECG) monitor recordings were found inversely associated with exposure to ambient PM originated from traffic and combustion sources [19,20]. Recent studies also reported the associations between HRV reduction and exposure to particles in smaller size including UFPs and particles in accumulation mode (with diameter less than 1 μm) [21,22], and among persons with cardiometabolic diseases or with metabolic syndromes [12,13,23,24]. However, the susceptibility of persons with cardiometabolic disease to the adverse effects of particulate matter is still uncertain, despite being biologically plausible.

Because of the increasing prevalence of diabetes and its association with an increasing risk of CVD, it is important to determine whether diabetes or impaired glucose tolerance may modify PM-HRV association. Some evidence suggests that ambient particles in small size fractions may play an important role in previously reported associations between traffic exposure and acute cardiovascular effects [4,25,26]. In this study, we assessed the role of exposure to particles in small size fractions 5-560 nm and black carbon (as a proxy for traffic related pollutant exposure) as cardiovascular risk factors in a group of subjects with diabetes or impaired glucose tolerance. We further examined if the association would be modified by diabetic state, obesity and gender.

## Results and discussion

### Results

The demographic and clinical characteristics of our study subjects are summarized in Table 1. The age of subjects ranged from 51 to 68 years old. There was no significant difference in gender ratio between type-2 diabetes (T2D) and impaired glucose tolerance (IGT) groups, or between obese and non-obese groups (*p*>0.1). No subject had severe cardiovascular diseases, except 68% of the subjects were with mild hypertension. All subjects with T2D were on regular medications (i.e. biguanides or sulfonylureas), and/or with insulin treatment; however, the subjects with IGT did not take regular medication, except one subject was taking biguanides regularly controlling for blood glucose level.

**Table 1:** **Demographic characteristics for IGT and T2D subjects**

	**IGT**	**T2D**
Number	17	36
Age (year)	60.1 (1.0)	59.7 (0.6)
BMI (kg/m ^2^)	25.3 (0.7)	26.7 (0.7)
Gender (Female/Male)	10/7	17/19
Weight (kg)	69.0 (1.8)	73.4 (2.0)
Height (cm)	165.2 (1.4)	165.4 (1.1)
Waist (cm)	92.2 (2.7)	92.8 (1.7)
Hip circumference (cm)	101.0 (2.0)	100.6 (1.3)
Waist/hip circumference	0.91 (0.01)	0.92 (0.01)
SDNN (ms)	119.6 (7.5)	108.6 (4.4)
rMSSD (ms)	23.8 (3.2)	19.8 (0.9)
LF (ms ^2^)	305.2 (62.5)	263.7 (25.5)
HF (ms ^2^)	160.7 (62.0)	95.9 (10.2)
Hypertension	8	28

Summary statistics of particle number concentrations (PNC) in six aggregated size fractions, BC, gaseous pollutants, and meteorologic parameters are summarized in Table 2. Within all the size fractions of particles, which are those of diameters from 5 nm to 560 nm, particles of size less than 200 nm dominated the number concentration, and ones of size 50-560 nm contributed the most of the surface concentration and the mass concentration (see Additional file 1).

**Table 2:** **Summary statistics of exposure and meteorologic parameters**

	**Mean (SD)**
PNC _5−560_ (1/cm ^3^)	20220 (11489)
PNC _5−10_ (1/cm ^3^)	1378 (1688)
PNC _10−20_ (1/cm ^3^)	4032 (4087)
PNC _20−50_ (1/cm ^3^)	6276 (4603)
PNC _50−100_ (1/cm ^3^)	5074 (2858)
PNC _100−200_ (1/cm ^3^)	2544 (1675)
PNC _200−560_ (1/cm ^3^)	272 (238)
BC (*μ*g/m ^3^)	4.09 (2.37)
NO _2_ (ppb)	32.52 (16.89)
CO (ppm)	0.61 (0.33)
SO _2_ (ppb)	6.53 (5.28)
O _3_ (ppb)	25.83 (18.7)
Temperature (°*C*)	22.62 (6.18)
RH (%)	67.86 (12.54)
Air pressure (bar)	1013.96 (6.42)

SD for standard deviation and IQR for interquatile range. The observations of all the pollutants and meteorologic parameters were obtained in 1 minute interval covering the whole period of each visit in April, June and September.

All metrics of concentrations of particles in the size fraction 50-100 nm and 100-200 nm were highly correlated with BC, NO_2_, and CO with correlation coefficients ranging from 0.55 to 0.78 (Table 3 and Additional file 1). The total number and surface area concentrations of particles were also highly correlated with BC and NO_2_. Particles in all size fractions of measurement were inversely correlated with O_3_, temperature and relative humidity (RH).

**Table 3:** **Correlations between number concentration of particles in each size fraction, and BC, gaseous pollutants and meteorologic parameters**

	**BC**	**NO**_**2**_	**CO**	**SO**_**2**_	**O**_**3**_	**T**	**RH**	**Air pressure**
PNC _5−560_	0.52	0.65*	0.45	0.48	-0.1	-0.13	-0.14	0.18
PNC _5−10_	-0.06	0.16	-0.05	0.09	0.08	-0.2	-0.15	0.34
PNC _10−20_	0.13	0.35	0.08	0.1	0.02	-0.08	-0.19	0.25
PNC _20−50_	0.35	0.54	0.36	0.38	-0.14	-0.04	-0.14	0.21
PNC _50−100_	0.75*	0.78*	0.65*	0.58	-0.28	-0.12	-0.07	0.04
PNC _100−200_	0.72*	0.7*	0.56	0.6*	-0.07	-0.2	-0.09	0.15
PNC _200−560_	0.37	0.36	0.3	0.31	-0.05	-0.19	0	0.18

T for temperature and * indicates correlation coefficients >0.6.

**Figure 1:**
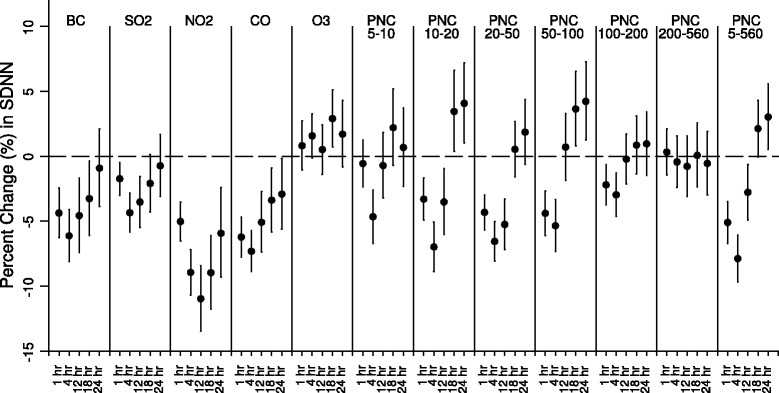
Results of single-pollutant mixed-effects model. It shows the changes in SDNN per IQR increase in different proceeding moving average exposures to ambient pollutants in the single-pollutant mixed-effects model.

In Figure 1, we presented the adjusted percent changes of standard deviation of all NN intervals (SDNN) per interquartile range (IQR) increase of number concentrations of particles in all size fractions, BC, and gaseous pollutants in various time windows of pollutant exposure, controlling age, gender, body mass index (BMI), time of day, day of week, temperature, RH and visit. Greater reductions in SDNN were associated with increase in exposure to particles in smaller size fractions <100 nm, followed by particles in 100-200 nm. Increased exposure to traffic-related pollutants BC, NO_2_ and CO, and combustion pollutant SO_2_, were also significantly associated with SDNN reductions. Overall, the inverse associations were the strongest for 4-hour average air pollutants exposure. No effect was observed for particles in size fraction of 200-560 nm and O_3_. Similar association patterns are observed for other HRV measures, including the root mean square of successive differences between adjacent normal cycles (rMSSD), low frequency (LF) (0.04-0.15 Hz) and high frequency (HF) (0.15-0.4 Hz), whereas the magnitude of reduction for frequency-domain measure LF and HF were greater (see Additional file 2).

To control the confounding effects of highly correlated pollutants, we compared the estimated effects of prior 4-hour average exposure to all size-fractioned particles, BC, O_3_, and NO_2_ in single- and two-pollutant models (Table 4). In two-pollutant models with adjustment for BC, the magnitude of effect estimates remained similar for number concentrations of particles of size 10 to 20 nm (PNC_10−20_), NO_2_, and CO as the magnitude in single-pollutant models. The magnitude of effects was reduced for PNC_20−50_, PNC_50−100_ and PNC_5−560_, whereas the effects became insignificant for PNC_100−200_ and PNC_200−560_. With adjustment for BC, the associations with surface area concentrations of particles (PSC), and mass concentrations of particles (PMC), remained significant for particles in size-fraction 10-20 nm and 20-50 nm only. In two-pollutant models with adjustment for NO _2_, the associations between SDNN and all measured pollutants, except BC, remained robust and consistent as assessed in single-pollutant models. We did not observe confounding effects of exposure to O_3_(results summarized in Additional file 3).

**Table 4:** **Percent change in SDNN per IQR increases in proceeding 4-hour moving average exposures to ambient pollutants estimated in single-, and two-pollutant mixed-effects models**

	**Single-pollutant**	**Two-pollutant**			
		**Adj. for BC**	**Adj. for NO**_**2**_	**Adj. for O**_**3**_	**Adj. for CO**
BC	-6.14 (-8.12,-4.11)	-6.14 (-8.12,-4.12)	1.09 (-1.72,3.98)	-5.18 (-7.31,-3)	0.07 (-2.7,2.91)
NO _2_	-8.98 (-10.72,-7.18)	-9.56 (-11.85,-7.2)	-8.97 (-10.72,-7.18)	-9.33 (-11.34,-7.27)	-6.43 (-8.64,-4.17)
CO	-7.33 (-8.91,-5.73)	-7.37 (-9.43,-5.25)	-7.05 (-8.95,-5.11)	-6.68 (-8.52,-4.81)	-4.22 (-6.19,-2.21)
SO _2_	-4.36 (-5.85,-2.86)	-2.91 (-4.66,-1.13)	-0.56 (-2.38,1.30)	-3.24 (-4.83,-1.62)	-1.25 (-3.02,0.55)
O _3_	1.55 (-0.13,3.27)	0.51 (-1.22,2.27)	1.55 (-0.14,3.28)	0.94 (-0.77,2.68)	-1.73 (-3.51,0.09)
PNC _5−560_	-7.89 (-9.69,-6.07)	-6.82 (-8.87,-4.72)	-7.73 (-9.57,-5.85)	-7.47 (-9.65,-5.24)	-4.54 (-6.82,-2.21)
PNC _10−20_	-7.0 (-8.88,-5.08)	-7.05 (-8.92,-5.14)	-7.21 (-9.14,-5.24)	-6.73 (-8.65,-4.77)	-6.18 (-8.1,-4.21)
PNC _20−50_	-6.57 (-8.07,-5.04)	-5.77 (-7.34,-4.17)	-6.36 (-7.92,-4.77)	-6.07 (-7.77,-4.33)	-4.32 (-6.07,-2.53)
PNC _50−100_	-5.37 (-7.34,-3.35)	-2.76 (-5.29,-0.16)	-5.65 (-7.69,-3.56)	-3.49 (-6.03,-0.89)	1.52 (-1.32,4.45)
PNC _100−200_	-2.98 (-4.63,-1.3)	2.53 (-0.29,5.42)	-2.53 (-4.26,-0.77)	0.3 (-1.84,2.49)	2.83 (0.71,4.99)
PNC _200−560_	-0.45 (-2.43,1.56)	5.15 (2.53,7.84)	0.09 (-1.96,2.18)	3.25 (0.97,5.59)	4.46 (2.22,6.75)

**Figure 2:**
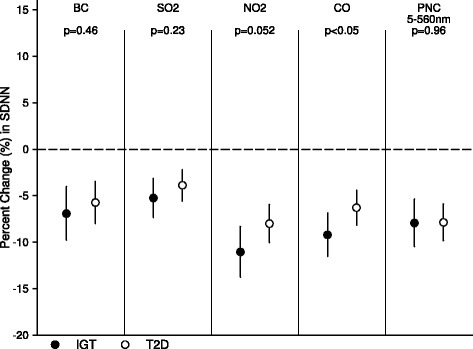
Stratified analysis by diabetic states. It shows estimates for adjusted percent changes in SDNN per IQR increase in 4-hour average exposures to different air pollutants in IGT and T2D groups.

**Figure 3:**
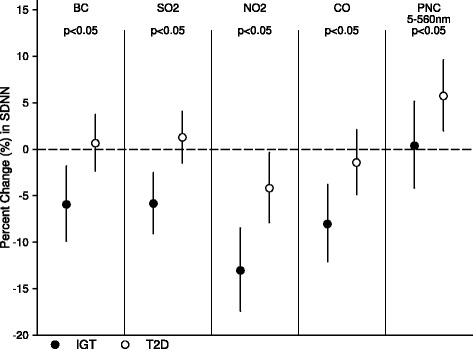
Stratified analysis by diabetic states. It shows estimates for adjusted percent changes in SDNN per IQR increase in 24-hour average exposures to different air pollutants in IGT and T2D groups.

Between subjects with T2D and in prediabetic state with IGT, effects assessed at 4- and 24-hour average exposure were presented in Figures 2 and 3. For prior 4-hour average air pollution exposure, we did not observe significant heterogeneity in effect estimates (with *p* values for interaction >0.05); however, effects in subjects with IGT were significantly stronger with prior 24-hour average exposure (with *p* values for interaction <0.05).

**Figure 4:**
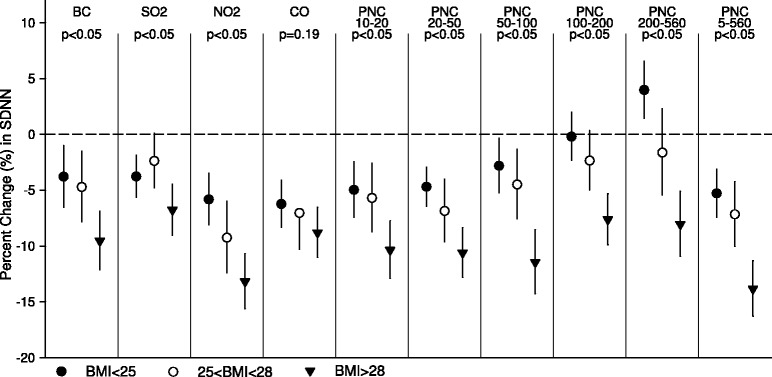
Stratified analysis by obesity. It shows estimates for adjusted percent changes in SDNN per IQR increase in 4-hour average exposures to different air pollutants in three groups stratified based on BMI.

Figure 4 shows the interaction effect of obesity on associations between SDNN and 4-hour average of air pollution exposure. Subjects with BMI>25 kg/m^2^ were found of greater SDNN reduction associated with exposure to air pollution, and the risk was even higher in subjects with BMI>28 kg/m^2^ (with *p* values for interaction <0.05). However, we did not find significant interaction of central adiposity in female nor in male subjects, though subjects with lower waist to hip ratio appeared in greater SDNN reduction associated with exposure to air pollution (with *p* values for interaction >0.05, data in Additional file 4). The correlation coefficients between obesity index BMI and central adiposity index waist to hip ratio (WHr) ranged 0.27 to 0.45 in our subjects.

**Figure 5:**
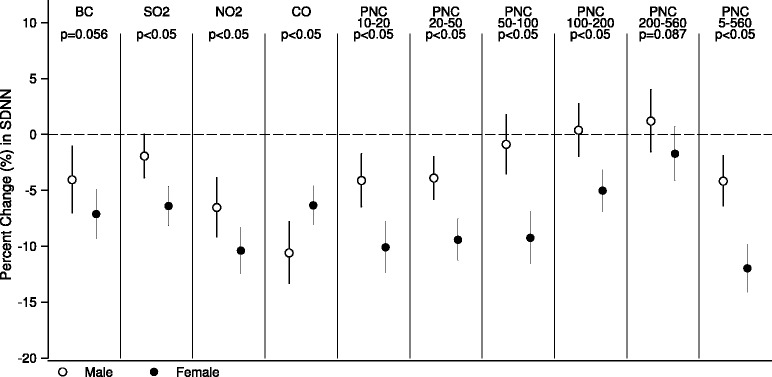
Stratified analysis by gender. It shows estimates for adjusted percent changes in SDNN per IQR increase in 4-hour average exposures to different air pollutants in male and female groups.

In the regression analyses of 4-hour average exposure stratified by gender, with controlling for same covariates in main models, we observed significant heterogeneity of gender (with *p* values for interaction <0.05) (Figure 5). Per IQR increase in 4-hour average of PNC _5−560_ (10,300 cm ^3^), we observed -12.0% (95% CI, -14.1% to -9.8%) in SDNN in female, and -4.2% (95% CI, -6.4% to -1.9%) in male, with *p* value for interactions <0.05. The modifiction effect of gender were significant when exposure to particles in size fraction <200 nm, NO_2_, SO_2_, or CO was considered, and marginally significant on BCexposure.

### Discussion

In our study, we observed significant inverse associations between autonomic dysfunctions in subjects with diabetes and impaired glucose tolerance and exposure to ambient particles in small size fractions (diameter less than 200 nm), BC, NO_2_ (traffic pollution proxy) and CO. The associations were stronger in subjects with obesity and in female. However, the interaction effect of diabetic states was not consistent over different averaging hours. Our findings suggest that possible cardiovascular effects associated with traffic pollution exposure may be mediated, in part, through altered autonomic function, among persons with diabetes or impaired glucose tolerance. Our findings have broad clinical and public health implications for air pollution induced cardiometabolic risks.

UFPs are an important component of combustion-related or secondary aerosol-related air pollution with large amount of toxic air pollutants and chemicals adsorbed on its surfaces. With its high lung deposition efficiency in the lungs, UFPs can enter into pulmonary interstitial sites and through circulation system reaching other target sites. The hypothesized intermediary pathophysiologic pathways associated with UFP exposures include release of proinflammatory mediators or vasculoactive molecules from lung-based cells, perturbation of systemic autonomic nerve system balance or heart rhythm, and potentially translocation of PM or PM constituents into circulation system [27]. Thus, we focused on examining cardiovascular risk associated with exposure to particles in small size fractions (between 5 to 560 nm) including UFPs, and traffic pollution markers (BC, NO_2_, and CO). Our overall results support the hypothesis that greater cardiovascular responses are associated with UFPs than with larger particles, and are consistent with findings of autonomic, vascular and thrombotic effects of UFPs inhalation observed in susceptible and healthy subjects [21,22,25,28,29].

However, the associations between UFPs exposures and acute changes in HRV reported in previous studies are not consistent and the directions and magnitudes of the associations varied across study locations. Chan et al. reported significant inverse relationship between hours prior average UFP exposures in HRV in a panel of healthy and elderly patients, with stronger effects in elderly subjects in Taiwan [30]. Weichenthal et al. reported significant reduction in HF and the proportion of NN50 divided by total number of NNs (pNN50) within hours immediately following UFP exposures in healthy cyclists in traffic in Ottawa, Canada [21].

Park et al. observed inverse association between ambient UFP exposures over 48-hour moving averages and HRV in elderly male subjects in Boston, USA, though the associations were not statistically significant [23].

In more recent studies, Schneider et al. did not observe significant associations between fixed-site ambient UFP measures and HRV over up to 47 lag hours in elderly men with coronary artery disease in Erfurt, Germany [14]; whereas Rich et al. reported decreased rMSSD associated with UFP exposures at significant level, but not with particles in accumulation mode (100-500 nm) and PM_2.5_, in a cardiac panel in rehabilitation in Rochester, USA [22]. Vora et al. lately reported that inhalation of elemental carbon ultrafine particles can alter heart rate and HRV in subjects with T2D [31]. It is possible that other pathophysiological factors such as activation of the renin-angiotensin system, oxidative stress, and inflammatory status in subjects with cardiovascular morbidities may have modulated the association between air pollution exposure and autonomic nervous system dysfunction.

Pathophysiologically, autonomic nervous system dysfunction also relates to insulin resistance [32,33], and abnormal glucose metabolism [34,35]. Park et al. reported significant HRV reduction in subjects with metabolic syndrome associated with exposure to 2-day average PM_2.5_ in six US communities in the Multi-Ethnic Study of Atherosclerosis (MESA) study, whereas no significant change was found among subjects without metabolic syndrome [13]. Whitsel et al. observed stronger inverse PM_10_-HRV associations in subjects with diabetes, and diabetic subjects of higher insulin and insulin resistance level were of statistically greater HRV reduction [12]. Recently, Min et al. observed a significant reduction in HRV related to exposure to carbon monoxide (CO) among persons with metabolic syndrome but not among persons without metabolic syndrome [36]. In our analysis, we examined the roles of diabetic state in modifying autonomic dysfunctions associated with ambient pollution. We observed significantly stronger inverse associations with 24-hour prior exposure to UFPs and traffic pollutants in subjects with IGT who did not receive regular medication to control for glucose level, but not in T2D subjects. However, with 4-hour prior exposure, the interaction of diabetic state between subjects with T2D and with IGT was not significant. The differential responses observed may be partially explained by insulin resistance or glucose metabolism altered by medication use in subjects.

As an important risk factors of metabolic syndrome, obesity is closely correlated with increased systemic inflammation and oxidative stress that are cardiovascular risk factors [37,38]. There is a growing body of evidence that T2D is a chronic inflammatory state aggravated by factors that promote inflammation at the level of vasculature and adipose tissue [39,40], and increased oxidative stress to adipocytes is central to the pathogenesis of cardiovascular disease in metabolic syndrome [41]. Air pollution may also promote T2D by increasing adipose inflammation and insulin resistance. Sun et al. recently found that PM_2.5_ exposure exaggerates whole-body insulin resistance and adiposity inflammation in mice [42]. Consistent with previous studies [20,24], obesity modified air pollution attributed cardiac autonomic function in our subjects. However, we did not observe interaction of abdominal fat accumulation. It seems plausible to hypothesize that adipose tissues located at different sites of body may have different metabolic properties in predisposing reduced skeletal muscle glucose utilization and insulin resistance, thus to pose differential mechanisms in regulation and/or impairments in sympathetic nervous system function associated with air pollution exposure.

Previous research suggested that gender could modify the cardiovascular risk and air pollution associations; however, the findings are heterogeneous [43,44]. Recent studies reported higher traffic pollution associated with cardiometabolic risks in females. Brook et al. found an association between modeled NO_2_ exposure and T2D prevalence among female patients, but not among male patients, of two respiratory health clinics in Canada [45]. A study by Kramer et al. supported the plausibility of oxidative stress and inflammation as a biological mechanism for the relation between air pollution and T2D, by showing that females with high C3c blood levels (a marker for subclinical inflammation) were more susceptible for particulate matter related excess risk of diabetes than were females with low C3c levels. This prospective study furthermore found a relation between traffic-related particulate matter and incident T2D among elderly women in Germany [46]. In addition, an American study found an association with distance to road among women, while no strong evidence of an association with particulate matter exposure was observed [47]. Consistent with our previous analysis [20], we observed greater HRV declines associated with exposures to traffic-related pollutants BC and gaseous pollutants in female elderly Chinese. Yet the prevalence of obesity and distribution of adipose tissue differ by age and gender which could modify cardiac responses pathophysiologically, the mechanisms for age, obesity and gender specific interactions of air pollution associated cardiovascular effects are not yet clear and deserve further investigation.

Several study limitations should also be noted in interpreting our findings. First, we only investigated subjects with T2D and IGT, and had no normal glucose tolerant or lean control subjects. Second, we did not collect individual data, such as insulin level and insulin resistance, homeostasis, systemic inflammation and oxidative stress, which might have limited our capability further examining the biological plausibility of diabetes in modifying UFPs and traffic pollution associated cardiovascular dysfunctions. Third, the fixed-location monitoring data was used in this study, and the lack of information on personal exposure to ambient pollution may have resulted in potential exposure misclassification errors and may have biased the effect estimates toward null. Fourth, we did not control subjects’ activity during observation, which may affect the results of frequency-domain measurement of HRV. And lastly, we did not obtain real-time minute-to-minute PM_2.5_ concentrations due to operational errors of the monitoring instruments installed in the monitoring laboratory. Thus we were not able to examine the commonly studied associations between autonomic dysfunction and exposure to criteria pollutant PM_2.5_, which has limited the generalization of our results in broader context of adverse effects of ambient fine particulates.

In summary, we observed autonomic dysfunction associated with increased exposure to ambient UFPs and traffic-related pollutants in previous hours among persons with diabetes or impaired glucose tolerance, and the magnitude of estimated effects were significantly stronger in obese persons and in females. Our results suggest that underlying metabolic condition may confer susceptibility of traffic pollution on the heart.

## Materials and methods

### Study design and subjects

This panel study was conducted in April, June and September of 2010. We recruited 53 elderly subjects with diagnosed type-2 diabetes or impaired glucose tolerance through an existing diabetes cohort followed by Luwan District Center for Disease Control (CDC) and Prevention in Shanghai. The diabetes cohort was designed to allow randomized or controlled evaluation of behavior and dietary modification on progression and risk factors of T2D. Since 2004, this cohort has enrolled approximately 4,000 individuals with diagnosed T2D or at risk, but free of clinically apparent cardiovascular diseases at baseline examination. At the enrollment, the disease state of each individual was identified by standardized medical record review and physician adjudication. During the follow-up, fasting plasma samples were measured every six months.

All the subjects were retired elderly living within one kilometer from Luwan CDC in central Shanghai. The information on subjects’ age, gender, weight, height, smoking status and medical history was obtained through administered baseline questionnaire interview during recruitment process. Our subjects received three repeated measurements of 24-hour ambulatory ECG during study period. The Institutional Review Board of Peking University Health Science Center approved study protocol and written consent form. Informed consent was obtained from each participant before the study participation.

### Individual metabolic abnormality measurement

Our subjects had fasting blood samples drawn and measured for plasma glucose, gycosylated hemoglobin, triglyceride, and cholesterol in routine follow-up in April and October in 2010. Each participant was scheduled arriving the check-up room in Luwan CDC at the same time of a weekday morning for all visits. At each visit, technical staff measured body weight, height, waist and hip circumferences, and blood pressure of each subject, and recorded 24-hour activity of the subjects prior to the visit through questionnaire survey. We used clinical cutoff points for diabetic states: T2D was defined as fasting blood glucose ≥ 126 mg/dL.

### HRV measures

After physical examination at each visit, trained personnel placed 7 bipolar leads of a ECG monitor on the participant, following standard protocol. HRV were derived from 24-h ECG recordings of subjects, under normal daily activity conditions, using a 3-channel Holter monitoring system (Model MGY H7, DM Software Inc., USA).

The ECG digital recordings were reviewed and processed by trained cardiologists. Mean heart rate and various measure of HRV were calculated for each 24-Hour session using PC-based software (Holter System Version 12.Net for Windows, DM Software). Each 5-minute segment of normal to normal intervals of the heart beat was used to calculate the HRV index that included time-domain measurements of SDNN and rMSSD, as well as frequency-domain measurements of LF and HF.

### Air pollution measures

Air pollution concentrations and meteorological data were monitored continuously using instruments equipped in a laboratory in Luwan CDC. The repeatability, accuracy, and predictive validity of the air pollution measurements conducted by the laboratory have been described previously [48].

Minute-to-minute number concentration of particles in size between 5 to 560 nm was monitored by a Fast Mobility Particle Sizer Spectrometer (FMPS Model 3091, Thermo, USA). Number-size distributions were converted to particle number concentrations, particle surface area concentrations, and particle mass concentrations, assuming spherical particles and 1 g/cm ^3^ as default density parameter. In this analyses, we calculated 5-minute mean concentration for particles in 5-10 nm, 10-20 nm, 20-50 nm, 50-100 nm, 100-200 nm, 200-560 nm size fractions. Total number, surface, and area concentrations of particles were computed as the sum of all fractions (5-560 nm).

We also measured real-time concentrations of BC, SO _2_, NO _2_, CO, and O _3_. Minute-to-minute BC concentration was measured by Multi Angle Absorption Photometer (MAAP Model 5012, Thermo Fisher Scientific Inc., USA). Minute-to-minute SO _2_, NO _2_, CO, and O _3_ concentrations were monitored by EC9800 Series Ambient Gas Analyzers (Eco Tech Pty. Ltd., Australia). Temperature (°C) and relative humidity (%) were measured by a Met One unit (Met One Instruments Inc., USA).

### Statistical analysis

We calculated descriptive statistics for each air pollution measurement. Spearman correlations were calculated across minute-to-minute data of air pollutants and meteorologic parameters. All HRV variables were logarithmically transformed using base 10 due to right skewed distributions.

We first built basic generalized linear mixed models with each HRV metric as the dependent variable. Based on the assessment of Akaikes information Criterion (AIC), we chose to model the temperature and RH using natural splines with degree of freedom up to 3, and modeled time of the day using quadratic polynomial functions. The first-order autoregressive model (AR1) was chosen to account for intra-subject correlation within each visit between repeated measures after comparison of several covariance structures, based on the criteria of minimizing AIC. The effect of uncontrolled features of subjects was modeled by random coefficient relying on subjects’ id. Models were also adjusted for potential confounders such as day of week, age, gender, BMI and visit.

Single-pollutant models were then developed by including pollutant variables to examine the associations between air pollution exposure and HRV measures. The effects of particles in all size-fractions and other pollutants under 1-, 4-, 12-, 18-, and 24-hour moving averages were examined. Due to relatively high correlation between concentration of particles and other air pollutants, we conducted two-pollutant analyses by including the second pollutant of prior 4-hour average exposure to examine the robustness of the estimates associated with particles.

To evaluate potential effect modification by status of disease and other factors, we conducted the analysis of SDNN with each air pollutant using the 4-hour averaging exposure, stratified by diabetic state (T2D vs. IGT), obesity (Group 1, BMI ≤25; Group 2, 25 ≤ BMI ≤28; Group 3, 28 ≤BMI), central adiposity (WHr), and gender, controlling the same covariates as in main models. We examined the heterogeneity in the association estimates across strata at the.05 significance level.

All effect estimates were expressed as percent change in each HRV variable for interquartile range (IQR) increase of the corresponding moving average of each pollutant, calculated as [10^(*β*×*IQR*)^−1]×100, with 95% confidence intervals (CI) {10^[*IQR*×(*β*∓1.96×*SE*)]^−1}×100, where *β* and *SE* are the estimated regression coefficient and its standard error. All analyses were conducted in SAS statistical software (Version 9.2; SAS Institute; Cary, NC).

## Additional files

Additional file 1
**Description and correlations.** This file contains 2 sheets. One is named ‘description_of_pollutants’, which is the extension of Table 2, with basic descriptive statistics of surface concentration (PSC) and mass concentration (PMC) of particles. The numbers after PSC or PMC indicate the size fraction of particles. Another sheet is named ‘correlations_among_pollutants’, which is an extension of Table 3, with correlations between surface or mass concentration of particles in each size fraction, and BC, gaseous pollutants and meteorologic parameters. T for temperature and * indicates correlation coefficients >0.6.

Additional file 2
**Per IQR change single pollutant.** This file contains 26 sheets. The name of the sheet gives the pollutant whose effect is shown in it, and the numbers after PNC, PMC or PSC indicate the size fraction of particles. Each sheet shows the percent change of all 5 HRV metrics, SDNN, rMSSD, LF, HF and LF/HF, in response to the IQR increases in 1-. 4-, 12-, 18-, and 24-hour moving averaged exposure to air pollutants. The column ‘Yvar’ indicates the HRV metric and the second column label the moving average. The column ‘Per IQR *↑*’ shows the percent change of the HRV metric, and the columns ‘95% CI-Low’ and ‘95% CI-High’ give the lower and upper bounds of 95% confidence interval.

Additional file 3
**Per IQR change two pollutant.** This file contains 1 sheet. It shows the effects of different metrics of particles on SDNN when another gaseous pollutant or BC is also included in the model. The time window for all pollutants is 4 hours prior to the HRV observation. The column ‘Confound Pol’ indicates which pollutant is included in the model to detect confounding effect. The column ‘Effect Pol’ indicates which pollutant is potentially confounded. The numbers after PNC, PMC or PSC indicate the size fraction of particles. The column ‘Per IQR *↑*’ shows the percent change of SDNN, and the columns ‘95% CI-Low’ and ‘95% CI-High’ give the lower and upper bounds of 95% confidence interval.

Additional file 4
**Per IQR change stratified.** This file contains 5 sheets. Each sheet shows the modification effect of a factor discussed in this study. The ratio of waist to hip circumference (WHr) is analyzed within each gender due to different classification criteria. We only considered the effect on SDNN and pollutants under prior 4-hour moving average for gender, BMI and WHr. For the modification effect of diabetic state, we analyzed both 4-hour and 24-hour time window. The group label is given in the first row of each sheet. The numbers after PNC, PMC or PSC indicate the size fraction of particles. The column ‘Per IQR *↑*’ shows the percent change of SDNN within each group, and the columns ‘95% CI-Low’ and ‘95% CI-High’ give the lower and upper bounds of 95% confidence interval.
